# Induced Pluripotent Stem Cells and Their Potential for Basic and Clinical Sciences

**DOI:** 10.2174/157340313805076278

**Published:** 2013-02

**Authors:** Lei Ye, Cory Swingen, Jianyi Zhang

**Affiliations:** 1 Department of Medicine, University of Minnesota Medical School, Minneapolis, Minnesota, USA; 2 Stem Cell Institute, University of Minnesota Medical School, Minneapolis, Minnesota, USA; 3 Department of Biomedical Engineering, University of Minnesota, Minneapolis, Minnesota, USA

**Keywords:** Cellular therapy, disease model, embryonic stem cells, induced pluripotent stem cells, reprogramm.

## Abstract

Induced pluripotent stem (iPS) cells, are a type of pluripotent stem cell derived from adult somatic cells. They have been reprogrammed through inducing genes and factors to be pluripotent. iPS cells are similar to embryonic stem (ES) cells in many aspects. This review summarizes the recent progresses in iPS cell reprogramming and iPS cell based therapy, and describe patient specific iPS cells as a disease model at length in the light of the literature. This review also analyzes and discusses the problems and considerations of iPS cell therapy in the clinical perspective for the treatment of disease.

## INTRODUCTION

Induced pluripotent stem (iPS) cells, are a type of pluripotent stem cell derived from adult somatic cells that have been genetically reprogrammed to an embryonic stem (ES) cell-like state through the forced expression of genes and factors important for maintaining the defining properties of ES cells.

Mouse iPS cells from mouse fibroblasts were first reported in 2006 by the Yamanaka lab at Kyoto University [1]. Human iPS cells were first independently produced by Yamanaka’s and Thomson’s groups from human fibroblasts in late 2007 [2, 3]. iPS cells are similar to ES cells in many aspects, including the expression of ES cell markers, chromatin methylation patterns, embryoid body formation, teratoma formation, viable chimera formation, pluripotency and the ability to contribute to many different tissues *in vitro*. 

The breakthrough discovery of iPS cells allow researchers to obtain pluripotent stem cells without the controversial use of embryos, providing a novel and powerful method to "de-differentiate" cells whose developmental fates had been traditionally assumed to be determined. Furthermore, tissues derived from iPS cells will be a nearly identical match to the cell donor, which is an important factor in research of disease modeling and drug screening. It is expected that iPS cells will help researchers learn how to reprogram cells to repair damaged tissues in the human body.

The purpose of this paper is to summarize the recent progresses in iPS cell development and iPS cell-based therapy, and describe patient specific iPS cells as a disease model, analyze the problems and considerations of iPS therapy in the clinical treatment of disease. 

## THE DEVELOPMENT OF iPS CELLS

The methods of reprogramming somatic cells into iPS cells are summarized in Table **1**. It was first demonstrated that genomic integration and high expression of four factors, Oct4/Sox2/Klf4/c-Myc or Oct4/Sox2/Nanog/LIN28 by virus, can reprogram fibroblast cells into iPS cells [1-3].  Later, it was shown that iPS cells can be generated from fibroblasts by viral integration of Oct4/Sox2/Klf4 without c-Myc [4]. Although these iPS cells showed reduced tumorigenicity in chimeras and progeny mice, the reprogramming process is much slower, and efficiency is substantially reduced. These studies suggest that the ectopic expression of these three transcription factors (Oct4/Klf4/Sox2) is required for reprogramming of somatic cells in iPS cells. 

Various growth factors and chemical compounds have recently been found to improve the induction efficiency of iPS cells. Shi *et al*., [5] demonstrated that small molecules, able to compensate for Sox2, could successfully reprogram mouse embryonic fibroblasts (MEF) into iPS cells. They combined Oct4/Klf4 transduction with BIX-01294 and BayK8644s and derived MEF into iPS cells. Huangfu *et al*., [6, 7] reported that 5-azacytidine, DNA methyltransferase inhibitor, and valproic acid, a histone deacetylase inhibitor, improved reprogramming of MEF by more than 100 folds. Valproic acid enables efficient reprogramming of primary human fibroblasts with only Oct4 and Sox2.

Kim *et al*. showed that mouse neural stem cells, expressing high endogenous levels of Sox2, can be reprogrammed into iPS cells by transduction Oct4 together with either Klf4 or c-Myc [19]. This suggests that endogenous expression of transcription factors, that maintaining stemness, have a role in the reprogramming process of pluripotency. More recently, Tsai *et al.*, [20] demonstrated that mouse iPS cells could be generated from the skin hair follicle papilla (DP) cell with Oct4 alone since the skin hair follicle papilla cells expressed endogenously three of the four reprogramming factors: Sox2, c-Myc, and Klf4. They showed that reprogramming could be achieved after 3 weeks with efficiency similar to other cell types reprogrammed with four factors, comparable to ES cells. 

 Retroviruses are being extensively used to reprogram somatic cells into iPS cells. They are effective for integrating exogenous genes into the genome of somatic cells to produce both mouse and human iPS cells. However, retroviral vectors may have significant risks that could limit their use in patients. Permanent genetic alterations, due to multiple retroviral insertions, may cause retrovirus-mediated gene therapy as seen in treatment of severe combined immunodeficiency [25]. Second, although retroviral vectors are silenced during reprogramming [26], this silencing may not be permanent, and reactivation of transgenes may occur upon the differentiation of iPS cells. Third, expression of exogenous reprogramming factors could occur. This may trigger the expression of oncogenes that stimulate cancer growth and alter the properties of the cells. Fourth, the c-Myc over-expression may cause tumor development after transplantation of iPS derived cells. Okita *et al.* [10] reported that the chimeras and progeny derived from iPS cells frequently showed tumor formation. They found that the retroviral expression of c-Myc was reactivated in these tumors. Therefore, it would be desirable to produce iPS cells with minimal, or free of, genomic integration. Several new strategies have been recently developed to address this issue (Table **1**). 

Stadtfeld *et al*. [16] used an adenoviral vector to transduce mouse fibroblasts and hepatocytes, and generated mouse iPS cells at an efficiency of about 0.0005%. Fusaki *et al*. [22] used Sendai virus to efficiently generate iPS cells from human skin fibroblasts without genome integration. Okita *et al*. [27] repeatedly transfected MEF with two plasmids, one carrying the complementary DNAs (cDNAs) of Oct3/4, Sox2, and Klf4 and the other carrying the c-Myc cDNA. This generated iPS cells without evidence of plasmid integration. Using a polycistronic plasmid co-expressing Oct4, Sox2, Klf4, and c-Myc, Gonzalez *et al*., [28] reprogrammed MEF into iPS cells without genomic integration. Yu *et al.* [29] demonstrated that oriP/EBNA1 (Epstein–Barr nuclear antigen-1)-based episomal vectors could be used to generate human iPS cells free of exogenous gene integration. The reprogramming efficiency was about 3–6 colonies/1 million somatic cells. Narsinh *et al*., [21] derived human iPS cells *via* transfection of human adipocyte stromal cells with a nonviral minicircle DNA by repeated transfection. This produced hiPS cells colonies from an adipose tissue sample in about 4 weeks. 

When iPS cells generated from either plasmid transfection or episomes were carefully analyzed to identify random vector integration, it was possible to have vector fragments integrated somewhere. Thus, reprogramming strategies entirely free of DNA-based vectors are being sought. In April 2009, it was shown that iPS cells could be generated using recombinant cell-penetrating reprogramming proteins [30]. Zhou *et al*. [30] purified Oct4, Sox2, Klf4 and c-Myc proteins, and incorporated poly-arginine peptide tags. It allows the penetration of the recombinant reprogramming proteins through the plasma membrane of MEF. Three iPS cell clones were successfully generated from 5x 104 MEFs after four rounds of protein supplementation and subsequent culture of 23–28 days in the presence of valproic acid. 

A similar approach has also been demonstrated to be able to generate human iPS cells from neonatal fibroblasts [31]. Kim *et al.* over-expressed reprogramming factor proteins in HEK293 cells. Whole cell proteins of the transduced HEK293 were extracted and used to culture fibroblast six times within the first week. After eight weeks, five cell lines had been established at a yield of 0.001%, which is one-tenth of viral reprogramming efficiency. Strikingly, Warren *et al.*, [24] demonstrated that human iPS cells can be derived using synthetic mRNA expressing Oct3/4, Klf4, Sox2 and c-Myc. This method efficiently reprogrammed fibroblast into iPS cells without genome integration.

Strenuous efforts are being made to improve the reprogramming efficiency and to establish iPS cells with either substantially fewer or no genetic alterations. Besides reprogramming vectors and factors, the reprogramming efficiency is also affected by the origin of iPS cells.

## CELL ORIGIN

A number of somatic cells have been successfully reprogrammed into iPS cells (Table **2**). Besides mouse and human somatic cells, iPS cells from other species have been successfully generated (Table **3**).

The origin of iPS cells has an impact on choice of reprogramming factors, reprogramming and differentiation efficiencies. The endogenous expression of transcription factors may facilitate the reprogramming procedure [19]. Mouse neural stem cells express higher endogenous levels of Sox2 and c-Myc than ES cells. Thus, two transcription factors, exogenous Oct4 together with either Klf4 or c-Myc, are sufficient to generate iPS cells from neural stem cells [19]. Ahmed *et al.* [14] demonstrated that mouse skeletal myoblasts endogenously expressed Sox2, Klf4, and c-Myc and can be easily reprogrammed to iPS cells. 

It is possible that iPS cells may demonstrate memory of parental source and therefore have low differentiation efficiency into other tissue cells. Kim *et al*. [32] showed that iPS cells reprogrammed from peripheral blood cells could efficiently differentiate into the hematopoietic lineage cells. It was found, however, that these cells showed very low differentiation efficiency into neural cells. Similarly, Bar-Nur *et al.* found that human β cell-derived iPS cells have the epigenetic memory and may differentiate more readily into insulin producing cells [33]. iPS cells from different origins show similar gene expression patterns in the undifferentiated state. Therefore, the memory could be epigenetic and are not directly related to the pluripotent status.

The cell source of iPS cells can also affect the safety of the established iPS cells. Miura *et al.* [54] compared the safety of neural differentiation of mouse iPS cells derived from various tissues including MEFs, tail-tip fibroblasts, hepatocyte and stomach. Tumorigenicity was examined. iPS cells that reprogrammed from tail-tip fibroblasts showed many undifferentiated pluripotent cells after three weeks of *in vitro* differentiation into the neural sphere. These cells developed teratoma after transplantation into an immune-deficient mouse brain. The possible mechanism of this phenomenon may be attributable to epigenetic memory and/or genomic stability. Pre-evaluated, non-tumorigenic and safe mouse iPS cells have been reported by Tsuji *et al.* [55]. Safe iPS cells were transplanted into non-obese diabetic/severe combined immunodeficiency mouse brain, and found to produce electrophysiologically functional neurons, astrocytes, and oligodendrocytes *in vitro*. 

The cell source of iPS cells is important for patients as well. It is important to carefully evaluate clinically available sources. Human iPS cells have been successfully generated from adipocyte derived stem cells [35], amniocytes [36], peripheral blood [38], cord blood [39], dental pulp cells [40], oral mucosa [41], and skin fibroblasts (Table **2**). The properties and safety of these iPS cells should be carefully examined before they can be used for treatment. 

Shimada *et al.* [17] demonstrated that combination of chemical inhibitors including A83-01, CHIR99021, PD0325901, sodium butyrate, and Y-27632 under conditions of physiological hypoxia human iPS cells can be rapidly generated from adipocyte stem cells *via* retroviral transduction of Oct4, Sox2, Klf4, and L-Myc. Miyoshi *et al*., [42] generated human iPS cells from cells isolated from oral mucosa *via* the retroviral gene transfer of Oct4, Sox2, c-Myc, and Klf4. Reprogrammed cells showed ES-like morphology and expressed undifferentiated markers. Yan *et al*., [40] demonstrated that dental tissue-derived mesenchymal-like stem cells can easily be reprogrammed into iPS cells at relatively higher rates as compared to human fibroblasts. Human peripheral blood cells have also been successfully reprogrammed into iPS cells [38]. Anchan *et al*. [36] described a system that can efficiently derive iPS cells from human amniocytes, while maintaining the pluripotency of these iPS cells on mitotically inactivated feeder layers prepared from the same amniocytes. Both cellular components of this system are autologous to a single donor. Takenaka *et al.* [39] derived human iPS cells from cord blood. They demonstrated that repression of p53 expression increased the reprogramming efficiency by 100-fold.

All of the human iPS cells described here are indistinguishable from human ES cells with respect to morphology, expression of cell surface antigens and pluripotency-associated transcription factors, DNA methylation status at pluripotent cell-specific genes and the capacity to differentiate *in vitro *and in teratomas. The ability to reprogram cells from human somatic cells or blood will allow investigating the mechanisms of the specific human diseases.

## iPS CELLS BASED CELL THERAPY

The iPS cell technology provides an opportunity to generate cells with characteristics of ES cells, including pluripotency and potentially unlimited self-renewal. Studies have reported a directed differentiation of iPS cells into a variety of functional cell types *in vitro*, and cell therapy effects of implanted iPS cells have been demonstrated in several animal models of disease.

### Cardiac Cells

A few studies have demonstrated the regenerative potential of iPS cells for three cardiac cells: cardiomyocytes, endothelial cells, and smooth muscle cells *in vitro* and *in vivo*. Mauritz [56] and Zhang [57] independently demonstrated the ability of mouse and human iPS cells to differentiate into functional cardiomyocytes *in vitro* through embryonic body formation. Rufaihah [58], *et al.* derived endothelial cells from human iPS cells, and showed that transplantation of these endothelial cells resulted in increased capillary density in a mouse model of peripheral arterial disease. Nelson *et al.* [59] demonstrated for the first time the efficacy of iPS cells to treat acute myocardial infarction. They showed that iPS cells derived from MEF could restore post-ischemic contractile performance, ventricular wall thickness, and electrical stability while achieving in situ regeneration of cardiac, smooth muscle, and endothelial tissue. Ahmed *et al.* [14] demonstrated that beating cardiomyocyte-like cells can be differentiated from iPS cells *in vitro*. The beating cells expressed early and late cardiac-specific markers. *In vivo* studies showed extensive survival of iPS and iPS-derived cardiomyocytes in mouse hearts after transplantation in a mouse experimental model of acute myocardial infarction. The iPs derived cardiomyocyte transplantation attenuated infarct size and improved cardiac function without tumorgenesis, while tumors were observed in the direct iPS cell transplantation animals. 

Strategies to enhance the purity of iPS derived cardiomyocytes and to exclude the presence of undifferentiated iPS are required. Implantation of pre-differentiation or guided differentiation of iPS would be a safer and more effective approach for transplantation. Selection of cardiomyocytes from iPS cells, based on signal-regulatory protein alpha (SIRPA) or combined with vascular cell adhesion protein-1 (VCAM-1), has been reported. Dubois *et al.* [60] first demonstrated that SIRPA was a marker specifically expressed on cardiomyocytes derived from human ES cells and human iPS cells. Cell sorting with an antibody against SIRPA could enrich cardiac precursors and cardiomyocytes up to 98% troponin T+ cells from human ESC or iPS cell differentiation cultures. Elliott *et al.* [61] adopted a cardiac-specific reporter gene system (NKX2-5eGFP/w) and identified that VCAM-1 and SIRPA were cell-surface markers of cardiac lineage during differentiation of human ES cells. 

### iPS Cells for Diabetes Mellitus

Regeneration of functional β cells from human stem cells represents the most promising approach for treatment of type 1 diabetes mellitus (T1DM). This may also benefit the patients with type 2 diabetes mellitus (T2DM) who need exogenous insulin. At present, technology for reprogramming human somatic cell into iPS cells brings a remarkable breakthrough in the generation of insulin-producing β cells.

Human ES cells can be directed to become fully developed β cells and it is expected that iPS cells could also be similarly differentiated. Stem cell based approaches could also be used for modulation of the immune system in T1DM, or to address the problems of obesity and insulin resistance in T2DM. 

Tateishi *et al.*, [62] demonstrated that insulin-producing islet-like clusters (ILCs) can be generated from the human iPS cells under feeder-free conditions. The iPS cell derived ILCs not only contain C-peptide positive and glucagon-positive cells but also release C-peptide upon glucose stimulation. Similarly, Zhang *et al*., [63] reported a highly efficient approach to induce human ES and iPS cells to differentiate into mature insulin-producing cells in a chemical-defined culture system. These cells produce insulin/C-peptide in response to glucose stimuli in a manner comparable to that of adult human islets. Most of these cells co-expressed mature β cell-specific markers such as NKX6-1 and PDX1, indicating a similar gene expression pattern to adult islet beta cells *in vivo*. 

Alipo *et al.* [64] used mouse skin derived iPS cells for differentiation into β-like cells that were similar to the endogenous insulin-secreting cells in mice. These β-like cells were able to secrete insulin in response to glucose and to correct a hyperglycemic phenotype in mouse models of both T1DM and T2DM after iPS cell transplant. A long-term correction of hyperglycemia could be achieved as determined by hemoglobin A1c levels. These results are encouraging and suggest that induced pluripotency is a viable alternative to directing iPS cell differentiation into insulin secreting β cells, which has great potential clinical applications in the treatment of T1DM and T2 DM.

Although significant progress has been made in differentiating pluripotent stem cells to β-cells, several hurdles remain to be overcome. It is noted in several studies that the general efficiency of *in vitro* iPS cell differentiation into functional insulin-producing β-like cells is low. Thus, it is highly essential to develop a safe, efficient, and easily scalable differentiation protocol before its clinical application. In addition, it is also important that insulin-producing b-like cells generated from the differentiation of iPS cells have an identical phenotype resembling that of adult human pancreatic β cells *in vivo*. 

### Neural Cells

Currently, the methodology of neural differentiation has been well established in human ES cells and shown that these methods can also be applied to iPS cells. Chambers *et al.* [65] demonstrated that the synergistic action of Noggin and SB431542 is sufficient to induce rapid and complete neural conversion of human ES and iPS cells under adherent culture conditions. Swistowsk *et al.* [66] used a completely defined (xenofree) system, that has efficiently differentiated human ES cells into dopaminergic neurons, to differentiate iPS cells. They showed that the process of differentiation into committed neural stem cells (NSCs) and subsequently into dopaminergic neurons was similar to human ES cells. Importantly, iPS cell derived dopaminergic neurons were functional as they survived and improved behavioral deficits in 6-hydroxydopamine-leasioned rats after transplantation. Lee *et al.* [67] provided detailed protocols for the step-wise differentiation of human iPS and human ES into neuroectodermal and neural crest cells using either the MS5 co-culture system or a defined culture system (Noggin with a small-molecule SB431542), NSB system. The average time required for generating purified human NSC precursors will be 2–5 weeks. The success of deriving neurons from human iPS cells provides a study model of normal development and impact of genetic disease during neural crest development.

Wernig *et al*., [68] showed that iPS cells can give rise to neuronal and glial cell types in culture. Upon transplantation into the fetal mouse brain, the cells differentiate into glia and neurons, including glutamatergic, GABAergic, and catecholaminergic subtypes. Furthermore, iPS cells were induced to differentiate into dopamine neurons of midbrain character and were able to improve behavior in a rat model of Parkinson's disease (PD) upon transplantation into the adult brain. This study highlights the therapeutic potential of directly reprogrammed fibroblasts for neural cell replacement in the animal model of Parkinson’s disease. 

Tsuji *et al*., [55] used pre-evaluated iPS cells derived for treatment of spinal cord injury. These cells differentiated into all three neural lineages, participated in remyelination and induced the axonal regrowth of host 5HT+ serotonergic fibers, promoting locomotor function recovery without forming teratomas or other tumors. This study suggests that iPS derived neural stem/progenitor cells may be a promising cell source for treatment of spinal cord injury.

Hargus *et al*., [69] demonstrated proof of principle of survival and functional effects of neurons derived from iPS cells reprogrammed from patients with PD. iPS cells from patients with Parkinson’s disease were differentiated into dopaminergic neurons that could be transplanted without signs of neuro-degeneration into the adult rodent striatum. These cells survived and showed arborization, and mediated functional effects in an animal model of Parkinson’s disease. This study suggests that disease specific iPS cells can be generated from patients with PD, which be used to study the PD development and *in vitro* drug screen for treatment of PD.

Reprogramming technology is being applied to derive patient specific iPS cell lines, which carry the identical genetic information as their patient donor cells. This is particularly interesting to understand the underlying disease mechanism and provide a cellular and molecular platform for developing novel treatment strategy.

## iPS CELLS AS A DISEASE MODEL

Human iPS cells derived from somatic cells, containing the genotype responsible for the human disease, hold promise to develop novel patient-specific cell therapies and research models for inherited and acquired diseases. The differentiated cells from reprogrammed patient specific human iPS cells retain disease-related phenotypes to be an *in vitro* model of pathogenesis (Table **4**). This provides an innovative way to explore the molecular mechanisms of diseases. 

Recent studies have reported the derivation and differentiation of disease-specific human iPS cells, including autosomal recessive disease (spinal muscular atrophy) [70], cardiac disease [71-75], blood disorders [13, 76], diabetes [77], neurodegenerative diseases (amyotrophic lateral sclerosis [78], Huntington’s disease [79]), and autonomic nervous system disorder (Familial Dysautonomia) [80]. Patient-specific cells make patient-specific disease modeling possible wherein the initiation and progression of this poorly understood disease can be studied. 

Human iPS cells have been reprogrammed from spinal muscular atrophy, an autosomal recessive disease. Ebert *et al*., [70] generated iPS cells from skin fibroblast taken from a patient with spinal muscular atrophy. These cells expanded robustly in culture, maintained the disease genotype and generated motor neurons that showed selective deficits compared to those derived from the patients' unaffected relative. This is the first study to show that human iPS cells can be used to model the specific pathology seen in a genetically inherited disease. Thus, it represents a promising resource to study disease mechanisms, screen new drug compounds and develop new therapies.

Similarly, three other groups reported their findings on the use of iPS cells derived cardiomyocytes (iPSCMs) as disease models for LQTS type-2 (LQTS2). Itzhaki *et al*., [72] obtained dermal fibroblasts from a patient with LQTS2 harboring the KCNH2 gene mutation and showed that action potential duration was prolonged and repolarization velocity reduced in LQTS2 iPS-CMs compared with normal cardiomyocytes. They showed that Ikr was significantly reduced in iPS-CMs derived from LQTS2. They also tested the potential therapeutic effects of nifedipine and the KATP channel opener pinacidil (which augments the outward potassium current) and demonstrated that they shortened the action potential duration and abolished early after depolarization. Similarly, Lahti *et al*., [73] demonstrated a more pronounced inverse correlation between the beating rate and repolarization time of LQTS2 disease derived iPS-CMs compared with normal control cells. Prolonged action potential is present in LQT2-specific cardiomyocytes derived from a mutation. Matsa *et al*., [74] also successfully generated iPS-CMs from a patient with LQTS2 with a known KCNH2 mutation. iPS-CMs with LQTS2 displayed prolonged action potential durations on patch clamp analysis and prolonged corrected field potential durations on microelectrode array mapping. Furthermore, they demonstrated that the KATP channel opener nicorandil and PD-118057, a type 2 IKr channel enhancer attenuate channel closing. 

LQTS3 has been recapitulated in mouse iPS cells [75]. Malan *et al.* [75] generated disease-specific iPS cells from a mouse model of a human LQTS3. Patch-clamp measurements of LQTS 3-specific cardiomyocytes showed the biophysical effects of the mutation on the Na+ current, withfaster recovery from inactivation and larger late currents than observed in normal control cells. Moreover, LQTS3-specific cardiomyocytes had prolonged action potential durations and early after depolarizations at low pacing rates, both of which are classic features of the LQTS3 mutation. 

Human iPS cells have been used to recapitulate diseases of blood disorder. Ye *et al.* [13] demonstrated that human iPS cells derived from periphery blood CD34+ cells of patients with myeloproliferative disorders, have the JAK2-V617F mutation in blood cells. Though the derived iPS cells contained the mutation, they appeared normal in phenotypes, karyotype, and pluripotency. After hematopoietic differentiation, the iPS cell-derived hematopoietic progenitor (CD34+/CD45+) cells showed the increased erythropoiesis and expression of specific genes, recapitulating features of the primary CD34+ cells of the corresponding patient from whom the iPS cells were derived. This study highlights that iPS cells reprogrammed from somatic cells from patients with blood disease provide a prospective hematopoiesis model for investigating myeloproliferative disorders.

Raya *et al*., [76] reported that somatic cells from Fanconi anaemia patients can be reprogrammed to pluripotency after correction of the genetic defect. They demonstrated that corrected Fanconi-anaemia specific iPS cells can give rise to haematopoietic progenitors of the myeloid and erythroid lineages that are phenotypically normal. This study offers proof-of-concept that iPS cell technology can be used for the generation of disease-corrected, patient-specific cells with potential value for cell therapy applications. 

Maehr *et al*., [77] demonstrated that human iPS cells can be generated from patients with T1DM by reprogramming their adult fibroblasts. These cells are pluripotent and differentiate into three lineage cells, including insulin-producing cells. These cells provide a platform to assess the interaction between β cells and immunocytes *in vitro*, which mimic the pathological phenotype of T1DM. This will lead to better understanding of the mechanism of T1DM and developing effective cell replacement therapeutic strategy. 

Lee *et al*., [80] reported the derivation of human iPS cells from patient with Familial Dysautonomia, an inherited disorder that affects the development and function of nerves throughout the body. They demonstrated that these iPS cells can differentiate into all three germ layers cells. However gene expression analysis demonstrated tissue-specific mis-splicing of *IKBKAP in vitro, *while neural crest precursors showed low levels of normal *IKBKAP *transcript. Transcriptome analysis and cell-based assays revealed marked defects in neurogenic differentiation and migration behavior. All these recaptured familial Dysautonomia pathogenesis, suggesting disease specificity of the with familial Dysautonomia human iPS cells. Furthermore, they validated candidate drugs in reversing and ameliorating neuronal differentiation and migration. This study illustrates the promise of disease specific iPS cells for gaining new insights into human disease pathogenesis and treatment.

Human iPS cells derived reprogrammed from patients with inherited neurodegenerative diseases, amyotrophic lateral sclerosis [78] and Huntington’s disease 79, have also been reported. Dimos *et al*., [78] showed that they generated iPS cells from a patient with a familial form of amyotrophic lateral sclerosis. These patient-specific iPS cells possess the properties of ES cells and were reprogrammed successfully to differentiate into motor neurons. Zhang *et al*., [79] derived iPS cells from fibroblasts of patient with Huntington’s disease. They demonstrated that striatal neurons and neuronal precursors derived from these iPS cells contained the same CAG repeat expansion as the mutation in the patient from whom the iPS cell line was established. This suggests that neuronal progenitor cells derived from Huntington’s disease cell model have endogenous CAG repeat expansion that is suitable for mechanistic studies and drug screenings.

Disease specific somatic cells derived from patient-specific human iPS cells will generate a wealth of information and data that can be used for genetically analyzing the disease. The genetic information from disease specific-iPS cells will allow early and more accurate prediction and diagnosis of disease and disease progression. Further, disease specific iPS cells can be used for drug screening, which in turn correct the genetic defects of disease specific iPS cells. 

## CONCLUSIONS 

iPS cells appear to have the greatest promise without ethical and immunologic concerns incurred by the use of human ES cells. They are pluripotent and have high replicative capability. Furthermore, human iPS cells have the potential to generate all tissues of the human body and provide researchers with patient and disease specific cells, which can recapitulate the disease *in vitro*. However, much remains to be done to use these cells for clinical therapy. A better understanding of epigenetic alterations and transcriptional activity associated with the induction of pluripotency and following differentiation is required for efficient generation of therapeutic cells. Long-term safety data must be obtained to use human iPS cell based cell therapy for treatment of disease. 

## Figures and Tables

**Table 1.** Reprogramming Factors

Reprogramming Factors	Cell Source	Vector	Other Conditions	References
Mouse Oct3/4, Sox2, Klf4, and c-Myc	MEF	Retrovirus		[1, 8-10]
	ME	Retrovirus	DNA methyltransferase and histone deacetylase inhibitors improve reprogramming efficiency	[6]
	Mouse BMMNCs	Retrovirus		[11]
	Mouse B lymphocytes	Retrovirus	Ectopic expression of C/EBPα or knockdown of Pax5	[12]
	Human cord blood or adult CD34+ cells	Retrovirus		[13]
	Mouse SkM	Retrovirus		[14]
	Mouse hepatocytes, and gastric epithelial cells	Retrovirus		[15]
	Mouse fibroblast and hepatocytes	Adenovirus		[16]
Mouse Oct3/4, Sox2, Klf4, and L-Myc	Human dipose-derived Stem cells	Retrovirus	Chemical inhibitors A83-01, CHIR99021, PD0325901, sodium butyrate, and Y-27632	[17]
Mouse Sox2, Klf4, and c-Myc	Pig fibroblast	Retrovirus	Oct4 is dispensable in generation of pig iPS.	[18]
Mouse Oct3/4, Sox2,and Klf4	Mouse tail and skin fibroblast	Retrovirus	Generates iPS without c-Myc transduction	[4]
Mouse Oct4 and Klf4 with small molecules	MEF	Retrovirus	BIX-01294 and BayK8644 facilities reprogramming	[5]
Mouse Oct4 and Klf4	Mouse neural stem cells	Retrovirus	Neural stem cells endogenously express Sox2	[19]
Mouse Oct4	Mouse hair follicle	Retrovirus	Skin dermal papilla cells express Sox2, c-Myc, and Klf4	[20]
Human Oct4, Sox2, Nanog, and LIN28	Human MSCs, fibroblast	Lentivirus	MSC derived from OCT4**knock-in ES/H1	[3]
	Human adipose cells	Nonviral	Minicircle	[ 21]
Human Oct3/4, Sox2, Klf4, and c-Myc	Human skin fibroblast	Retrovirus	Slc7a1 modified fibroblasts	[2]
	Human skin fibroblast	Sendai virus		[22]
Human Oct3/4, Sox2, Klf4, and Glis1	Mouse and human fibroblast	Retrovirus		[23]
Human Oct4 and Sox2	Human fibroblast	Retrovirus	Valproic acid improves reprogramming efficiency	[7]
Human Klf4, c-Myc, Oct4, Sox2 mRNA	Human fibroblast	mRNA		[24]

MEF: mouse embryonic fibroblast; SkM: Skeletal myoblast

**Table 2.** Cell Origin of iPS Cells

Cell Source	Vector	Reprogramming Factors
MEF [1]	Retrovirus	Mouse Oct3/4, Sox2, Klf4, and c-Myc
Mouse adult bone marrow mononuclear cells [11]	Retrovirus	Mouse Oct3/4, Sox2, Klf4, and c-Myc
Mouse B lymphocytes [12]	Retrovirus	Mouse Oct3/4, Sox2, Klf4, and c-Myc
Mouse skeletal myoblasts [14]	Retrovirus	Mouse Oct3/4, Sox2, Klf4, and c-Myc
Mouse tail tip fibroblasts and skin fibroblasts [4]	Retrovirus	Oct3/4, Sox2,and Klf4
Mouse neural stem cells [19]	Retrovirus	Oct4 together with either Klf4 or c-Myc
Mouse liver and stomach cells [15]	Retrovirus	Oct3/4, Sox2, Klf4, c-Myc
Mouse fibroblast and hepatocyte [16]	Adenovirus	Oct3/4, Sox2, Klf4, c-Myc
Mouse pancreatic islet beta cells [34]	Lentivirus	Oct3/4, Sox2, Klf4, and c-Myc
Mouse hair follicles: [20]	Retrovirus	Mouse Oct4
Mouse and human fibroblast [23]	Retrovirus	Oct3/4, Sox2, Klf4, and Glis1
Mouse or human ADS cells [35]	Retrovirus	mouse Oct3/4, Sox2, Klf4, and c-Myc
Mouse and human amniocytes [36]	Retrovirus	Human=Oct4, Sox2,Klf4, c-Myc, mouse= Klf4, c-Myc
Human ADS [17, 21, 37]	Retrovirus Nonviral minicircle	Mouse Oct4, Sox2, Klf4, and L-Myc Oct4, Sox2, Nanog and LIN28
Human CD34+ blood cells [13,38]	Retrovirus	Oct3/4, Sox2, Klf4, and c-Myc
Human CD34+ cord blood cells [39]	Retrovirus	Oct4, Sox2, Klf4, and c-Myc
Human dental tissue cells [40]	Retrovirus	Oct4, Sox2, Klf4, c-Myc or Lin28,Nanog, Oct4, Sox2
Human skin fibroblast [2]	Retrovirus	human Oct3/4, Sox2, Klf4, and c-Myc
Human somatic cells [3]	Lentivirus	Oct4, Sox2, Nanog, and LIN28
HUVEC [41]	Retrovirus	Oct3/4, Sox2, Klf4, and c-Myc
Human oral mucosa fibroblasts [42]	Retrovirus	Oct3/4, Sox2, Klf4, and c-Myc

ADS= Adipose-derived stem cells HUVEC= human umbilical vein endothelial cells

**Table 3.** Species of iPS Cells

Species	Tissue	Vector	Reprogramming Factors
Canine	Adult fibroblast [43]	Retrovirus	Human Oct4, Sox2, Klf4, and c-Myc
Equine	Equine Fetal Fibroblast [44]	Plasmid	Oct4, Sox2, Klf4 and c-Myc
Monkey	Fetal or adult fibroblast [45, 46]	Retrovirus	Monkey Oct4, Sox2, Klf4, and c-Myc
Pig	Adult ear fibroblast [47]	Plasmid	Oct4, Sox2, Klf4, c-Myc
	Mesenchymal stem cell [48]	Lentivirus	human Oct4, Sox2, Nanog, KLF4, LIN28, and c-MYC
	Fetal fibroblast [49]	Lentivirus	human Oct4, S0x2, Klf4, and c-Myc
Rabbit	Liver and stomach cells [50]	Lentivirus	human c-Myc, Klf4, Sox2, and Oct4
Rat	Rat embryonic fibroblasts [51]	Lentivirus	Mouse Oct4, Klf4, and Sox2
Sheep	Fetal fibroblast [52]	Lentivirus	Mouse Oct3/4, Klf4, and Sox2
	Adult fibroblast [53]		Oct4, Sox2, c-Myc, Klf4, Nanog, etc.

**Table 4.** Disease Modeling Using Human iPS Cells

Disease	Cell Origin	Reference
Amyotrophic lateral sclerosis	Dermal fibroblast	[78]
Blood disorders	Human CD34+ blood cells	[13]
	Dermal fibroblast	[76]
Diabetes	Skin fibroblast	[77]
Familial Dysautonomia	Fibroblast	[80]
Huntington’s disease	Fibroblast	[79]
LEOPARD syndrome	Fibroblast	[81]
Long-QT syndrome1	Fibroblast	[71]
Long-QT syndrome2	Fibroblast	[72-74]
Long-QT syndrome3	MEF cell model	[75]
Spinal muscular atrophy	Fibroblast	[70]
Timothy syndrome	Fibroblast	[82, 83]
